# Rapid and High Seed Germination and Large Soil Seed Bank of *Senecio aquaticus* in Managed Grassland

**DOI:** 10.1100/2012/723808

**Published:** 2012-01-04

**Authors:** Matthias Suter, Andreas Lüscher

**Affiliations:** ^1^Swiss Grassland Society AGFF, Reckenholzstrasse 191, 8046 Zurich, Switzerland; ^2^ETH Zurich, Department of Environmental Sciences, Institute of Integrative Biology, Universitätstrasse 16, 8092 Zurich, Switzerland; ^3^Agroscope Reckenholz-Tänikon Research Station ART, Forage Production and Grassland, Reckenholzstrasse 191, 8046 Zurich, Switzerland

## Abstract

*Senecio aquaticus*, regionally a Red List species in Europe, has become increasingly abundant in agricultural grassland of medium to high management intensity in Switzerland, Southern Germany, and Austria in recent years, where it is a threat for animal and human health due to its toxicity. In this study, we investigated the seed ecology of *S. aquaticus* to help protection of the species in relic populations while improving its control when abundant in managed grassland. Germination percentages of fresh ripe seeds of *S. aquaticus* were on average 68% in 2008, but only 45% in 2010, indicating yearly variation. Germination was generally fast: ten days after the onset of the tests, often more than 45% of all seeds had germinated. When covered with a soil layer of 5 mm, germination was only 16% compared to 63% in full light. Seeds buried in the soil for one and two years showed a germination of 78%, significantly higher than that of fresh ripe seeds, thus suggesting a stimulating effect of cold-wet stratification on germination and long seed survival in the soil. In grasslands with established populations of *S. aquaticus*, the number of germinable seeds of the species ranged from 361 to 1875 m^−2^ in topsoil (0–10 cm) with an average of 1139 m^−2^. The large seed bank and the rapid and high germination of *S. aquaticus* suggest that allowing seed formation is important for its preservation in relic populations. With respect to agricultural grassland, strategies to control the species should initially target hindering seed production and dispersal.

## 1. Introduction

*Senecio aquaticus* Hill (marsh ragwort) [1], regionally a Red List species in Europe (e.g., Northern Germany, the Netherlands), has mainly become rare due to habitat degradation [2]. Interestingly, the species has been able to spread into agricultural grassland of Central Europe (Southern Germany, Eastern Austria, Switzerland) in recent years, thus attracting increasing attention [3]. *S. aquaticus * can pose a threat to animal and human health [4] because it contains pyrrolizidine-alkaloids (PAs) that are toxic for cattle and other livestock [5–7] and PAs can be transferred into milk [8].

According to Sebald et al. [9], *S. aquaticus * often grows in drained and cultivated wetland that is now lightly fertilised and mown once or twice per year, but the species can also exist at sites with moderate to high management intensity [10] than ten individuals per m^2^ [11]. *S. aquaticus * is reported to be a biennial forb and individuals produce several 100 seeds per year; the seeds; the seeds bear a pappus that allows for wind dispersial [12]. The substantial seed production could lead to a soil seed bank at sites where the species has persisted for many years. Gaps seem to be a key factor facilitating invasion and propagation of *S. aquaticus * in agricultural grassland [10], indicating that light availability may be a determining factor in seed germination of the species. 

While the occurrence of *S. aquaticus * in relic population should be sustained, further spread into agricultural grassland must be prevented [4]. Measures for control of *S. aquaticus * in managed grassland can include herbicides [13], but with a high soil seed bank, *S. aquaticus * is likely to continuously germinate following chemical application [11]. Sustainable and efficient long-term regulation of *S. aquaticus * must focus on management practices that consider the ecological behaviour of the species. However, ecologically relevant features of *S. aquaticus * such as the germination behaviour or the seed bank characteristics are widely unknown.

Fast and high seed germination of *S. aquaticus * could partially explain the species' success in colonising new sites in grasslands because rapid germination can increase a species' competitive advantage over others [14]. Moreover, as population dynamics of grassland species are linked to their soil seed bank [15, 16], the existence of a high soil seed bank for *S. aquaticus * might further shed light on its high local abundance in Central Europe. To evaluate such questions, we collected fresh ripe seeds of *S. aquaticus * from mature plants in the Northern Prealps of Switzerland and brought them to germination in a series of standardised tests under varying conditions. In addition, we assessed seed survival in the soil by burying fresh ripe seeds with mesh bags for one and two years in natural grasslands before bringing them to germination. Finally, we took samples of the topsoil and of a deeper soil layer to assess the size of the natural soil seed bank. Such data on the seed ecology of *S. aquaticus * can help protect relic populations in nature conservation areas, while improving strategies for the species' control in agricultural grassland.

## 2. Materials and Methods

### 2.1. Study Sites and Seed Material

Three locations within the centre of the geographical distribution of *S. aquaticus * in Switzerland were selected for sampling and experimentation, hereafter named Kriens I (810 m a.s.l.), Kriens II (800 m a.s.l.), and Rothenthurm (910 m a.s.l.). Each of the selected sites was permanent grassland containing more than five thousand flowering individuals of the species distributed over an area of approximately one hectare, and populations of *S. aquaticus * were older than five years. The grasslands were selected so as to provide a diverse range of management and soil conditions: applied plant-available Nitrogen was 30–130 kg ha^−1^ year^−1^, defoliation frequency was 2–5 year^−1^, soil clay content was 21–26%, and soil organic carbon ranged from 4 to 27%.

For the evaluation of germination characteristics of fresh ripe seeds, individuals of *S. aquaticus*, including stalks, leaves, and flowers, were collected from the three sites at the end of anthesis in August 2008 and in late July 2010. The plant and seed material was spread out and stored at room temperature for a minimum of three weeks to ensure smooth and consistent drying of the seeds before they were cleaned from the brackets and the pappus. The mean seed mass of 6000 seeds sampled evenly from across the three sites (3 × 2000 seeds) was 0.326 × 10^−3^ g seed^−1^ in 2008, but only 0.225 × 10^−3^ g seed^−1^ in 2010.

### 2.2. Soil Samples and Their Preparation

Soil samples for testing the seed bank were cored from topsoil (0–10 cm) in May 2007 and from a deeper layer (20–30 cm) in late April 2008. At each of the three sites, nine subareas were identified by stratified random sampling; on each subarea, twenty-one soil cores were pooled into one sample of 0.8 litres. Litter on top of the soil cores was removed and samples were kept in dark conditions at 2°C until further processing.

Soil samples were prepared following ter Heerdt et al. [17]: the soil was concentrated by washing with a jet of water through a coarse (3 mm mesh width) and a fine sieve (0.35 mm), thus removing coarse and very fine soil material, roots, and vegetative parts. The second mesh was fine enough to catch the seeds of *S. aquaticus * and most other species at the sites. The volume of the concentrated samples was approximately 0.16 litres.

### 2.3. Germination Procedure

The germination tests took place in a shaded glasshouse that had light (natural and supplementary) between 06 : 00 and 22 : 00. Temperatures were generally maintained at 24°C from 06 : 00 to 22 : 00 and 18°C from 22 : 00 to 06 : 00, but during warm days, temperature sometimes rose to 28°C. Mean relative air humidity was 65%.

The fresh ripe seed material was split into replicates of 200 seeds, which were mixed with 0.16 litres of substrate containing 37% peat, 32% compost, 18% expanded clay, 3% clay, and 10% sand. Such substrate for germination provided more natural conditions than filter paper and was similar to the soil from the cores.

**Figure 1:**
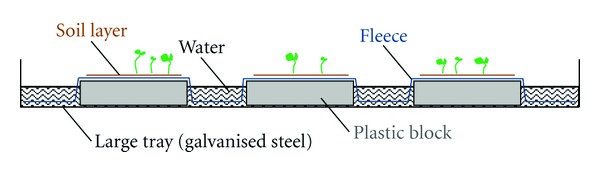
Details of the technical facility for seed germination to ensure constant moisture conditions. The large tray was 50 cm × 70 cm in size and the plastic blocks were each 20 cm × 20 cm.

The germination procedure proposed by ter Heerdt et al. [17] was further improved to ensure consistent moisture during germination. The substrates mixed with seeds were spread on top of fleece in quadrates of 180 mm × 180 mm, thereby creating layers of 5 mm thickness. Each piece of fleece (3 mm thick) covered three plastic blocks (20 cm × 20 cm) and was submerged into water in a large tray (Figure 1). The water level was, on average, 3 cm below the surface of the soil layer and was adjusted every second or third day. This method enabled slight, but constant moisture up-take into the soil, thus matching the conditions where *S. aquaticus * generally occurs. Unless otherwise stated, the soil layer on the fleece was thoroughly crumbled twice to provide light to all seeds, and germinated seedlings were removed when the cotyledons were completely unfolded. All tests were run for seven to eight weeks; subsequent to this period, no or only marginal germination was observed.

Preceding tests for dormancy in *S. aquaticus * using Gibberellic Acid (GA) revealed that GA had no significant effect on germination (*P* > 0.1 in each of three independent tests). Therefore, we concluded that potential dormancy in *S. aquaticus * cannot be broken by GA as applied in our tests, and this treatment was not considered further (details see Appendix).

### 2.4. Topic I: Germination Characteristics of Fresh Ripe Seeds and Light Effect

Fresh ripe seeds of the three sites were tested in October 2008, and germination was determined at days ten and 56. This test was repeated with seeds sampled in 2010, but germination was recorded every second or third day to highlight dynamics over time. In 2010, in addition to the standard treatment of seeds mixed with soil that was crumbled twice, a second treatment assessed the effect of light on germination by spreading seeds on top of the soil layer. Finally, in contrast to the latter treatment, a third test was run in which seeds were completely covered by a soil layer of 5 mm without any soil crumbling (only two sites tested due to limited seed material).

### 2.5. Topic II: Germination and Survival of Seeds Buried in the Soil

Seeds sampled in 2008 were mixed with substrate and collected in polyester mesh bags with a volume 0.18 litres (mesh size 190 *μ*m, Lanz-Anliker AG, Switzerland). Twenty mesh bags were buried, in an even distribution over each of the three sites, to a depth of 18 cm and precisely marked in late November 2008. All remaining seeds sampled in 2008 were stored in the fridge at 6°C. In October 2009, ten mesh bags per site were dug out and brought to germination alongside ten replicates per site from the refrigerated seeds. This procedure was repeated in October 2010 with the remaining buried mesh bags and refrigerated seeds, thus assessing the effect of cold-wet stratification on germination under natural conditions, in which soils of the sites were deeply wetted and covered by snow during winter.

### 2.6. Topic III: Size of Soil Seed Bank in Natural Grasslands

The test for seeds in the topsoil (0–10 cm) was performed in July 2007. Seedlings of *S. aquaticus*, other forbs (dicots), and grasses (monocots) were counted and removed once they could be distinguished at a size of 2-3 cm. The test for seeds in the deeper soil layer (20–30 cm) was performed in May 2008, but only seedlings of *S. aquaticus * were identified.

### 2.7. Data Analysis

Data were analysed with generalised linear models, and inference was based on treatment contrasts from these regressions. For analysis of fresh ripe seed germination, we used the logit-link function assuming a binomial error distribution, from which we considered the response to be restricted between 0 and 1 (1 = 100% germination). For data on the soil seed bank, the log-link function was used, that is, assuming a Poisson error distribution, as highly nonnormal residuals were expected due to spatial patterns in the soil seed bank. Large trays (Figure 1) were always modeled as a block variable; however, no block effect has been observed in any analyses. All analyses were carried out using the statistics software R [18].

## 3. Results

### 3.1. Topic I: Rapid and High Germination of Fresh Ripe Seeds and Distinct Light Effect

**Figure 2:**
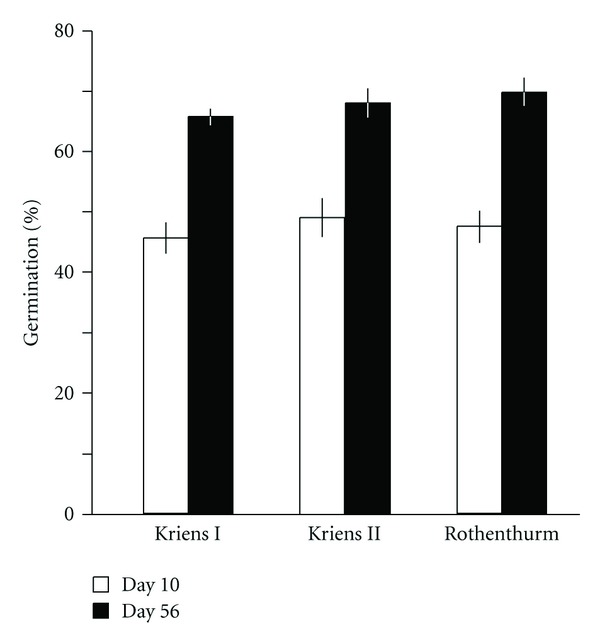
Germination percentages of fresh ripe seeds of *Senecio aquaticus* sampled in 2008. Displayed are means ± SE (*n* = 10) at day 10 and day 56 of an eight-week germination period. For germination, seeds were mixed with substrate that was thoroughly crumbled twice to provide light to all seeds.

**Figure 3:**
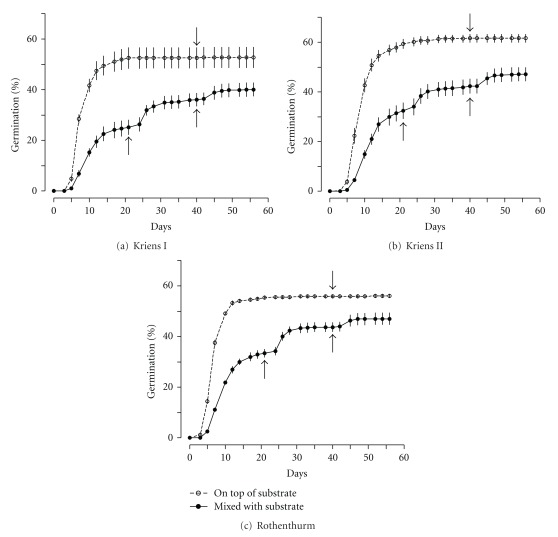
Germination percentages of fresh ripe seeds of *Senecio aquaticus* sampled in 2010; displayed are means ± SE (*n* = 5). For germination, seeds either lay on top of or were mixed with substrate that was spread in 5 mm thick layers. At noted dates (↓, ↑) substrate was thoroughly crumbled to provide light to all seeds.

Germination rates of seeds of *S. aquaticus * sampled in 2008 were high: the first seedlings were visible by day 5, and by day 10, more than 45% of the seeds from all three sites had germinated (Figure 2). The final germination percentage was, on average, 68% (SE ± 1%, seeds of this test were mixed with soil). No significant differences in germination among the sites were observed, neither at day ten (*P* = 0.694) nor at the end of the test (*P* = 0.392). Final germination percentages of *S. aquaticus * seeds sampled in 2010 were on average 45% (±2%) in the treatment in which seeds were mixed with soil, indicating variation in germination from year to year (compare Figures 2 and 3). The lower germination in 2010 fit with the lower mean seed mass in 2010 compared to 2008 (see Section 2). 

However, when seeds in 2010 were spread in full light on top of the soil, final average germination was 58% (±2%) (Figure 3); the difference in germination to seeds mixed with soil being highly significant (*P* < 0.001). Moreover, seeds on top of the soil layer germinated significantly faster from the onset: when compared at day ten, 44% (±1%) of seeds in full light were germinated compared to only 17% (±1%) when mixed with soil (*P* < 0.001 for the difference). These results suggest that the absence of light may prevent seeds of *S. aquaticus * from germinating, a hypothesis that was further tested by the treatment which permanently covered seeds with a soil layer of 5 mm. In this test, covered seeds germinated on average with 16% (±2%), while seeds in full light germinated with 63% (±5%; *P* < 0.001 for the difference; no figure shown).

### 3.2. Topic II: High Survival of Seeds Buried in the Soil

**Figure 4:**
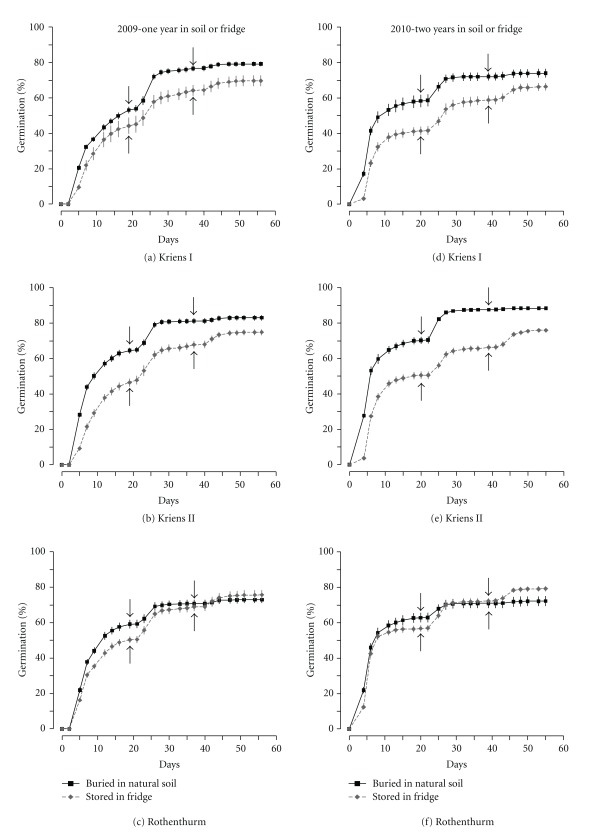
Germination percentages of seeds of *Senecio aquaticus* stored in the fridge and buried in natural soil; displayed are means ± SE (*n* = 10). For germination, seeds were mixed with substrate that was spread in 5 mm thick layers. At noted dates (↓, ↑) substrate was thoroughly crumbled to provide light to all seeds.

Seeds sampled in 2008 and buried in the soil for one year had an average germination percentage of 78% (±1%) (Figures 4(a)–4(c)). This value was significantly greater than that for seeds stored in the fridge (73%  ± 1%, *P* = 0.008 for the difference) and for fresh ripe seeds (*P* = 0.001, compare Figures 2 and 4). Similar results were observed for seeds buried in the soil (79%  ± 2%) or stored in the fridge (74%  ± 1%; Figures 4(d)–4(f)) for two years. Seeds buried in the soil for two years also germinated very fast, with an average rate of 58% (±2%) by day ten.

### 3.3. Topic III: Large Seed Bank in the Topsoil

The soil seed bank of *S. aquaticus * ranged from 361 to 1875 m^−2^ (Table 1; all values scaled to 10 cm topsoil) with an overall mean of 1139 germinable seeds m^−2^ (SE ± 196). As a comparison, the mean number of germinable seeds of forbs was 6713 m^−2^ (SE ± 1143.8; all other forb species pooled), while the mean number of seeds of grasses was 30755 m^−2^ (SE ± 3733.4; all grass species pooled).

Almost no germinable seeds of *S. aquaticus * were found in the deeper soil layer (20–30 cm); only at Kriens II, 40 germinable seeds m^−2^ were observed. In all three sites, the difference between the germination in the upper and lower soil depths was highly significant (*P* < 0.001, Table 1).

## 4. Discussion

### 4.1. Germination Characteristics of Fresh Ripe Seeds

Germination of *S. aquaticus * was fast and high: after ten days, more than 45% of the seeds had germinated and the total germination increased up to 70% after eight weeks. Rapid germination enhances a species' chances to achieve a competitive advantage over others due to size-asymmetric competition for light, which is a decisive growth factor in temperate grasslands [19]. Initial size differences between seedlings are enhanced over time with asymmetric competition. Thus, *S. aquaticus*, which often germinates in gaps of established vegetation [10], might profit from initial size differences, capturing space and resources at the expense of other species' seedlings. Such competitive behaviour at early growing stages can directly relate to the abundance of mature plants in vegetation [14].

Germination percentages of *S. aquaticus * were generally higher than those of species of temperate grasslands, particularly when compared to those of wet meadow species [20–22]. In our tests, fresh ripe seeds of *S. aquaticus * revealed increased germination when exposed to full light, while a cover of only a few millimetres of soil prevented most seeds from germination. High and rapid germination under full light is a feature of a species that favours open patches and disturbance [23] and partially explains the success of *S. aquaticus * in colonising gaps in grasslands. Combined with the high number of pappus-bearing seeds, such fast and high germination clearly increases the species' chances to persist and expand its populations into regularly mown or grazed agricultural grassland. 

### 4.2. Soil Seed Bank

With an average of over 1000 germinable seeds m^−2^ in the topsoil, the seed bank of *S. aquaticus * was large when compared to other species in similar grasslands [24, 25]. The total number of germinable seeds of all species in the soil ranged between 20000 and 60000 m^−2^, with the greatest share coming from monocots, in agreement with a recent report [26]. Thus, approximately 3% of the topsoil seeds were derived from *S. aquaticus*. This finding corresponds to the unimpaired, high germination of seeds that were buried in the soil for two years (Figure 4) and the substantial reduction of germination in the absence of light (Figure 3). In combination, the results suggest a persistent seed bank with high seed survival of *S. aquaticus * in the soil.

At first glance, large persistent seed banks and high germination of fresh ripe seeds appear contradictory. However, we argue that this apparent contrast in *S. aquaticus * can be resolved by accounting for its germination response to light. Growing mostly in established wet grasslands, only a small fraction of the annual seed production of *S. aquaticus * can germinate due to severe light interception by the existing vegetation. The majority of seeds may become covered by litter or incorporated into the topsoil by the activity of mice or earthworms [27, page 137]. In the absence of full light, these seeds become dormant and contribute to the formation of a seed bank, behaviour that has been demonstrated for many other species of temperate fens [28]. In our study, soil samples were collected in late spring and litter was removed to ensure that seeds originated from the soil. We therefore conclude that, despite high germination of fresh seeds, a large seed bank can be formed, particularly where old and established populations of *S. aquaticus * exist.

Thompson and Grime [29] have distinguished two types of persistent seed banks: the first type arises when most of the seeds germinate after they are released, but a small portion of the seeds fail to do so and become incorporated into the soil. The second type of persistent seed bank is observed when few of the seeds germinate immediately following dispersal, and the species maintains a seed bank that is large in relation to the annual seed production. In *S. aquaticus*, depending on yearly variation in seed production and the fraction of seeds able to germinate, the seed bank may have a pattern that corresponds rather to the first than the second type. The absence of seeds of *S. aquaticus * in the deeper soil layer reflects that our samples originated from sites that have not been ploughed for decades, a status that holds for most habitats of *S. aquaticus * in Central Europe.

Survival of seeds in the soil often follows a negative exponential relationship [27, page 149], meaning that seeds have a constant rate of death. Based on this relation and an estimated death rate of 0.3 per year, seed survival of the well-known *Senecio jacobaea* L. (common ragwort) has been calculated to be greater than ten years [30, 31], and such seed survival may also hold for *S. aquaticus*, given the close relationship between these two species that even allows for hybrid formation [32]. In our study, rather than reduction, we found an increase in germination when seeds were buried in the soil for one and two years. First, this suggests a slight innate dormancy, as some fresh ripe seeds that did not germinate in 2008 germinated in the following year. Second, this result implies a positive effect of cold-wet stratification on germination of *S. aquaticus*, because soils had temperatures around zero degrees Celsius during the winter. Such conditions correspond to experiments that demonstrate the positive effect of cold-wet stratification on germination of wetland species [20, 33]. Third, and most importantly, the sustained high germination of *S. aquaticus * is a support for high seed survival in the soil; the species still had an average germination of approximately 80% after two years, with no detectable death over this time. It must be assumed that some seed mortality will appear after three or four years. Given the observed 80% germination after two years, and assuming a negative exponential decay and a (probably too high) death rate of 0.2 per year, the estimated average germination of seeds in the soil from *S. aquaticus* would still be approximately 10% after ten years.

### 4.3. Implications for Practice

As a biennial forb, *S. aquaticus * must regularly regenerate from seeds to maintain its population size, and our results show that *S. aquaticus * exhibits features that complement such a life cycle: germination of fresh ripe seeds was high, and seeds buried in the soil became dormant and successfully germinated when brought back to light. This implies that a cutting regime allowing the species' seed production at least once per year, gap formation in meadows, or disturbance of the uppermost soil layer would promote populations of *S. aquaticus * in nature conservation areas. Because its germination is fast and high, such treatment would allow *S. aquaticus * to outperform other wetland species that often exhibit very low germination [20, 33, 34]. If seed formation of *S. aquaticus * is possible over several years, a seed bank would develop that further enhances the species persistence and survival.

With respect to agricultural grassland, strategies against the further spread of *S. aquaticus * can target hindrance of seed formation and dispersal. When the species first appears in managed grassland and only few individuals are present, pulling or digging will effectively inhibit further spread. However, the seed ecology of *S. aquaticus * suggests that control might become difficult when fully established in large populations. Such establishment is accompanied by large seed banks, and, in association with high seed survival and rapid and high germination, the species will permanently germinate and re-grow when soil disturbance occurs. Inclined sites, where *S. aquaticus * often occurs in the mountain regions of Switzerland, Southern Germany, and Austria, are particularly susceptible to well-established populations of *S. aquaticus * because gap formation by cattle grazing or mechanical harvest cannot be avoided. Under such conditions, control of fully established populations of *S. aquaticus * in agricultural grassland may last for several years until the seed bank is depleted.

## Figures and Tables

**Table 1:** Means (±SE; *n* = 9) of germinable seeds of *Senecio aquaticus*, forbs, and grasses m^−2^ in the soil at three grasslands with established populations of *S. aquaticus* located in the centre of the geographical distribution of the species in Switzerland.

Site	*S. aquaticus*		Forbs	Grasses
Soil depth	0–10 cm	20–30 cm^‡^	0–10 cm	0–10 cm
Kriens I	361 (±115.0)	0	12486 (±2242.7)	46639 (±5746.6)
Kriens II	1875 (±387.0)	42 (±41.7)	2597 (±441.3)	29653 (±4287.6)
Rothenthurm	1181 (±257.8)	0	5056 (±951.1)	15972 (±5048.5)

^‡^ *n* = 3 for soil depth 20–30 cm.

## Appendix

### Test of Dormancy of Seeds

Germination of viable seeds evaluated in the laboratory can be low due to dormancy [35, 36]. Measures to break seed dormancy include cold-wet stratification or the application of Gibberellic acid (GA), the latter being a convenient approach in germination tests [37, 38]. In this study, we tested three cases for dormancy in *S. aquaticus * with GA: fresh ripe seeds, seeds stored for two years in the fridge at 6°C, and seeds buried in the soil with mesh bags. Each test consisted of a total of 30 replicates (15 with and without GA, with five replicates per treatment from each of three sites). Germination conditions were as described in the core text. For GA treatment, 40 mL GA-H_2_O solution was poured over the soil layers (GA : H_2_O solution ratio was 0.35 g : 1 litre), while the control received the same volume of water only. In all cases, GA had no positive effect on germination; the probability of error for the difference between treatment and control was *P* = 0.128 with fresh ripe seeds (germination with GA was 4% less than without), *P* = 0.527 with seeds stored in the fridge (germination with GA was 2% less than without), and *P* = 0.839 with seeds buried in the soil (germination with GA was 2% less than without; *P*-values from treatment contrasts based on a generalised linear model). Therefore, we concluded that potential dormancy in *S. aquaticus * cannot be broken by GA as applied in these tests.
